# Serum BDNF as an integrative neuro-immunometabolic marker in adolescent polycystic ovary syndrome: divergent dietary and inflammatory associations across weight phenotypes

**DOI:** 10.3389/fnut.2026.1830074

**Published:** 2026-05-21

**Authors:** Małgorzata Mizgier, Dorota Formanowicz, Veronica Sansoni, Grażyna Jarząbek-Bielecka, Giovanni Lombardi, Witold Kędzia, Justyna Opydo-Szymaczek

**Affiliations:** 1 Department of Sports Dietetics, Faculty of Health Sciences, Poznan University of Physical Education, Poznan, Poland; 2 Chair and Department of Medical Chemistry and Laboratory Medicine, Poznan University of Medical Sciences, Poznan, Poland; 3 Laboratory of Experimental Biochemistry and Advanced Diagnostics, I.R.C.C.S. Ospedale Galeazzi-Sant’Ambrogio, Milano, Italy; 4 Division of Gynecology, Department of Gynecology, Center of General Sexology and Sexology of Developmental Age, Poznan University of Medical Sciences, Poznan, Poland; 5 Department of Athletics, Strength and Conditioning, Poznań University of Physical Education, Poznań, Poland; 6 Department of Pediatric Dentistry, Poznan University of Medical Sciences, Poznan, Poland

**Keywords:** adolescence, antioxidant defence, BDNF, diet, inflammation, insulin sensitivity, omega-3 fatty acids, oxidative stress

## Abstract

**Background:**

Polycystic ovary syndrome (PCOS) in adolescence is characterized by hormonal imbalance, low-grade inflammation, oxidative stress, and metabolic dysfunction. Brain-derived neurotrophic factor (BDNF) regulates energy homeostasis and neuroimmune signaling and may act as a neuroimmunometabolic integrator in PCOS.

**Methods:**

Forty adolescent females with PCOS (26 normal weight and 14 with overweight and obesity) were evaluated. Serum BDNF, omega-3 fatty acids (EPA, DHA), uric acid, metabolic and hormonal indices, and total antioxidant capacity (T-AOC) were measured. Dietary intake and physical activity were assessed using validated tools.

**Results:**

Serum BDNF concentrations did not differ between BMI phenotypes. After FDR correction, significant dietary associations were observed only in normal-weight participants, including energy, protein, fat, and unsaturated fatty acids. No dietary associations remained significant in patients with overweight and obesity. Across both phenotypes, BDNF was positively associated with IL-1β, MDA, T-AOC, EPA, and uric acid. IL-6, TNF-*α*, and DHA remained significant only in normal-weight participants. No associations were found with CRP, lipid profile, sex hormones, or glucose–insulin parameters.

**Conclusion:**

Circulating BDNF was associated with dietary, inflammatory, and oxidative parameters, with limited evidence for endocrine associations. These findings suggest that BDNF may reflect an integrated neuroimmunometabolic profile in adolescent PCOS and should be considered exploratory, requiring confirmation in longitudinal studies.

## Highlights

- Serum BDNF in adolescents with PCOS was associated with diet quality, inflammatory activity, and oxidative–antioxidative balance rather than adiposity alone.
- Divergent associations observed between normal-weight and higher-BMI phenotypes suggest phenotype-dependent differences in neuroimmunometabolic regulation.
- These findings suggest that serum BDNF may represent a candidate indicator of linked nutritional, inflammatory, redox, and neuroendocrine processes in adolescent PCOS.

## Introduction

Polycystic ovary syndrome (PCOS) is one of the most prevalent endocrine disorders in adolescent patients and is characterized by a multifaceted interplay between neuroendocrine, metabolic, inflammatory, and lifestyle-related mechanisms (1, 2). During adolescence, these processes occur in a period of dynamic physiological maturation, rendering this population particularly vulnerable to early disturbances in insulin sensitivity, oxidative stress, low-grade inflammation, and disordered eating behaviors (3–5). Our previous study also indicates that improving diet quality may beneficially modulate immunometabolic profiles in adolescent PCOS (6).

Brain-derived neurotrophic factor (BDNF) is traditionally recognized for its central role in neurogenesis, synaptic plasticity, and cognitive function (7, 8). However, accumulating evidence indicates that BDNF also acts as a peripheral metabolic regulator, influencing appetite, mitochondrial energetics, lipid utilization, and muscle-driven metabolic flexibility (9–12). BDNF shows dual responsiveness to inflammatory and oxidative signals: pro-inflammatory cytokines, including interleukin-1β (IL-1β), interleukin-6 (IL-6), and tumor necrosis factor-*α* (TNF-α) and oxidative stress markers can induce compensatory increases in BDNF expression, which may function as a neuroprotective and metabolic resilience mechanism (8, 13, 14).

The regulation of circulating BDNF is further shaped by dietary intake and nutritional quality. Diets rich in protein, monounsaturated and polyunsaturated fatty acids (MUFA and PUFA), and omega-3 fatty acids have been linked to increases in circulating BDNF (7, 15–19), whereas high intake of saturated fats or overall poor diet quality may attenuate neurotrophic responses (20, 21). Moreover, physical activity is a potent stimulant of muscle-derived BDNF, although effects vary with intensity, age, and training status (22–24).

Despite these insights, no previous study has examined circulating BDNF in adolescents with PCOS in relation to diet composition, omega-3 fatty acids, inflammatory markers, oxidative–antioxidative balance, uric acid, insulin sensitivity, sex hormones, and habitual physical activity simultaneously. Importantly, whether these associations differ between normal-weight and the phenotype with overweight or obesity—an axis of substantial metabolic heterogeneity—remains unknown.

Given the emerging evidence that circulating BDNF represents a composite signal of central and peripheral origins, we hypothesized that BDNF in adolescent PCOS reflects an integrated neuroimmunometabolic response rather than adiposity alone, and that regulatory patterns differ according to weight phenotype. The present study, therefore, aimed to (a) compare serum BDNF levels across body mass index (BMI)-defined phenotypes and (b) investigate phenotype-specific associations between BDNF and dietary, metabolic, inflammatory, oxidative, hormonal, and physical activity parameters.

## Materials and methods

### Study design and participants

This cross-sectional study is part of an ongoing project investigating metabolic, inflammatory, and neuroendocrine markers in adolescent girls with PCOS, with methodological details previously described in Mizgier et al. (25). Participants aged 14–18 years were recruited consecutively and diagnosed according to the Rotterdam criteria adapted for adolescents. Based on BMI, they were stratified into two phenotypes: normal weight (*n* = 26, non-Ov/Ob) and overweight or obesity (*n* = 14, Ov/Ob). Overweight and obesity were classified using age- and sex-specific BMI-for-age z-scores according to the World Health Organization (WHO) growth reference for children and adolescents aged 5–19 years, as described previously in our cohort (2).

Exclusion criteria comprised chronic systemic disorders other than PCOS, acute infection, the use of hormonal or anti-inflammatory medication within three months prior to sample collection, and the use of nutritional supplements or other agents with potential metabolic, antioxidant, or anti-inflammatory effects during the same period. Examples of such conditions included diabetes mellitus, thyroid disease, autoimmune disorders, chronic inflammatory conditions, and other clinically significant endocrine or metabolic diseases. Participants with diagnosed psychiatric disorders or active eating disorders were also excluded.

Written informed consent was obtained from all participants and their parents or legal guardians. The study protocol was approved by the local Bioethics Committee (approval no. 553/18, add. 416/22, add. 684/25).

### Clinical assessment and sample collection

All participants underwent clinical evaluation during a 2-day hospital stay, including menstrual and clinical assessment, physical examination, and transabdominal ultrasound, as previously described (2). Venous blood samples (2 × 7.5 mL) were collected in the morning, between 7:00 and 8:00, after an overnight fast, during the early follicular phase (days 3–5). Serum was aliquoted and stored at −80 °C until analysis.

### Biochemical analyses

#### BDNF and uric acid

Serum BDNF and uric acid concentrations were measured in duplicate using ELISA kits (Sunred Bio, China) following the manufacturer’s instructions. The assay sensitivity was 0.05 ng/mL for BDNF and 6.256 μmol/L for uric acid. Results were calculated using the MyAssays online tool.^1^

#### Omega-3 fatty acids (EPA and DHA)

Serum eicosapentaenoic acid (EPA) and docosahexaenoic acid (DHA) were quantified using commercial ELISA kits (DRG MedTek Sp. z o.o., Poland). The analytical sensitivity and measuring range were as follows: EPA – sensitivity 0.125 ng/mL, range 0.2–30 ng/mL; DHA – sensitivity 0.08 ng/mL, range 0.095–20 ng/mL.

### Oxidative and antioxidant markers

Total antioxidant capacity (T-AOC) was determined using a colorimetric assay, following standardized spectrophotometric procedures. Malondialdehyde (MDA) and inflammatory cytokines [IL-1β, IL-6, TNF-*α*, C-reactive protein (CRP)] were previously described in Mizgier et al. (25).

### Hormonal and metabolic parameters

Follicle-stimulating hormone (FSH), luteinizing hormone (LH), estradiol and prolactin were measured using electrochemiluminescence immunoassays (ECLIA; Elecsys, Roche Diagnostics GmbH, Mannheim, Germany), while testosterone, dehydroepiandrosterone sulfate (DHEA-S), sex hormone-binding globulin (SHBG), fasting glucose, and insulin were previously described in Mizgier et al. (25). Procedures for FSH, LH, and estradiol followed the methodologies described in Wendland et al. (26). Indices of insulin resistance and sensitivity were calculated as described in Mizgier et al. (2). Insulin sensitivity was additionally assessed using the standard Quantitative Insulin Sensitivity Check Index (QUICKI), calculated as 1/[log(fasting insulin) + log(fasting glucose)].

### Dietary intake assessment

Dietary intake was assessed using three-day food records and analyzed for energy, macronutrients, fatty acids (saturated fatty acids (SFA), MUFA, PUFA), total sugars, sucrose, and fiber, according to protocols previously described (25). Participants received standardized verbal and written instructions on how to complete the 3-day food records, and the records were reviewed with a researcher to improve completeness and plausibility.

### Physical activity assessment

Habitual physical activity was evaluated using the International Physical Activity Questionnaire – Short Form (IPAQ-SF) based on validated procedures (27–29). Activity levels were categorized into low, moderate, or high according to standard metabolic equivalent of task (MET)-minutes per week thresholds. The IPAQ-SF was administered according to validated procedures.

### Statistical analysis

All statistical analyses were performed using MedCalc Statistical Software version 23.3.7 (MedCalc Software Ltd., Ostend, Belgium). Normality of the distribution was assessed using the Shapiro–Wilk test. Normally distributed variables were expressed as mean ± standard deviation (SD), and non-normal variables as median [Q1–Q3]. Between-group differences were tested using Student’s t-test (with Welch’s correction when applicable), Mann–Whitney U test, one-way analysis of variance ANOVA, or Kruskal–Wallis test as appropriate. Correlations were evaluated using Pearson’s r for normally distributed variables and Spearman’s *ρ* for non-normally distributed variables. A *p*-value < 0.05 was considered statistically significant. To control for multiple testing in correlation analyses, False Discovery Rate (FDR) correction was applied using the Benjamini–Hochberg procedure. Adjusted *p*-values (q-values) were calculated separately within predefined biological domains and within BMI-defined subgroups.

## Results

### Study cohort

**Figure 1:**
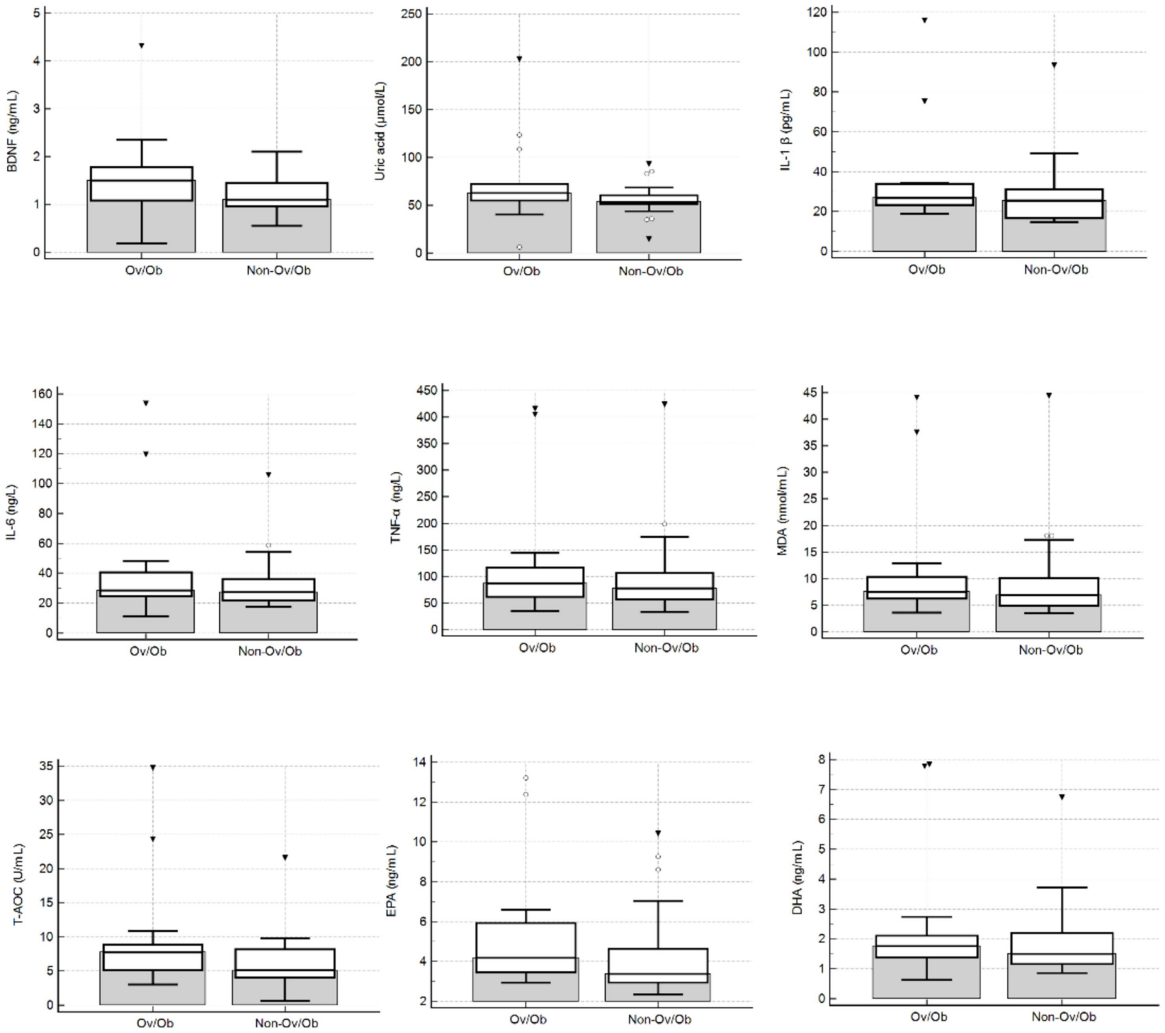
Comparison of oxidative stress, inflammatory, and neurotrophic markers between Ov/Ob and Non-Ov/Ob participants with PCOS. Data are presented as boxplots showing the median (horizontal line), interquartile range (box), and whiskers extending to the minimum and maximum values, excluding outliers. Circles represent mild outliers (values exceeding 1.5 × IQR), and triangles indicate extreme outliers (values exceeding 3 × IQR).

The study cohort’s characteristics are presented in Table 1. No significant differences were observed between patients with overweight and obesity, and normal-weight in terms of age and height. As expected, weight and BMI were significantly higher in the Ov/Ob group (*p* < 0.0001 for both comparisons). Fasting glucose and insulin levels were also significantly higher in the Ov/Ob group compared to Non-Ov/Ob patients (*p* = 0.0286 and *p* = 0.0091, respectively). Serum BDNF levels did not differ significantly between the two phenotypes. Selected biomarkers representing key components of the hypothesized neuro–immuno–metabolic axis were additionally visualized to facilitate phenotype-based comparison (Figure 1). No statistically significant differences were observed between groups (Mann–Whitney U test, all *p* > 0.05).

**Table 1:** Baseline characteristics and serum BDNF levels in adolescents with PCOS according to BMI phenotype.

Parameters	Ov/Ob (*n* = 14)	Non-Ov/Ob (*n* = 26)	*p* value
Age (years)	15.5 [14; 17]	16 [16; 17]	0.1133*
BMI (kg/m^2^)	30.89 ± 3.94	19.67 ± 2.49	<0.0001**
Height (m)	1.67 ± 0.05	1.66 ± 0.06	0.3178***
Weight (kg)	86.54 ± 10.65	53.97 ± 7.72	<0.0001***
Fasting glucose (mg/dL)	90.65 ± 7.89	86.08 ± 4.84	0.0286**
Fasting insulin (μU/ml)	19.56 [11.30; 30.61]	11.19 [8.29; 14.87]	0.0091*
BDNF ng/ml	1.50 [1.08; 1.78]	1.10 [0.96; 1.45]	0.1735*

Data are presented as median (Q1–Q3) or mean±SD, as appropriate. Ov/Ob, participants with overweight or obesity; Non-Ov/Ob, participants with normal weight; BMI, body mass index; BDNF, brain-derived neurotrophic factor. The *p*-values marked in bold were statistically significant. *Mann–Whitney test, **T-test (Welch’s correction), ***T-test.

### BDNF and dietary intake of energy and macronutrients

Distinct dietary patterns were observed across BMI phenotypes. In the normal-weight group, several positive associations between serum BDNF and dietary variables were identified. After FDR correction, significant correlations remained for total energy intake, total protein, plant protein, total fat, and unsaturated fatty acids, including MUFA and PUFA, with the strongest association observed for PUFA.

In contrast, in the Ov/Ob group, no dietary variables remained significantly associated with BDNF after FDR correction. Detailed correlation coefficients, *p*-values, and FDR-adjusted q-values for both groups are presented in Table 2.

**Table 2:** Correlations between serum BDNF and dietary, inflammatory, oxidative, and metabolic parameters in adolescent participants with PCOS according to BMI phenotype.

	Ov/Ob (*n* = 14)			Non-Ov/Ob (*n* = 26)		
	*r*	*p* value	*q*	*r*	*p* value	*q*
Dietary variable						
Energy (kcal)	−0.16 *	0.5838	0.6830	0.45**	0.0213	**0.0426**
Proteins (g)	−0.52*	0.0596	0.4698	0.54**	0.0044	**0.0264**
Fats (g)	−0.35*	0.2269	0.6489	0.46**	0.0168	**0.0403**
Carbohydrates (g)	−0.10*	0.7479	0.7479	0.15**	0.4651	0.5581
Dietary fibres (g)	−0.14*	0.6261	0.6830	0.38*	0.0585	0.0878
Plant proteins (g)	−0.40*	0.1559	0.6236	0.50**	0.0094	**0.0282**
Animal proteins (g)	−0.22*	0.4456	0.6684	0.41**	0.0380	0.0651
Total Sugars (g)	−0.27*	0.3499	0.6489	0.02**	0.9365	0.9372
Sucrose (g)	−0.27*	0.3581	0.6489	−0.02**	0.9372	0.9372
SFA (g)	−0.49*	0.0783	0.4698	0.21*	0.3147	0.4196
MUFA (g)	−0.26*	0.3785	0.6489	0.50**	0.0093	**0.0282**
PUFA (g)	−0.18*	0.5371	0.6830	0.64*	0.0005	**0.0060**
Inflammatory, oxidative, metabolic variable						
IL-1β (pg/mL)	0.69*	0.0065	**0.0146**	0.55*	0.0037	**0.0048**
IL-6 (ng/L)	0.52*	0.0586	0.0753	0.67*	0.0002	**0.0004**
TNF-α (ng/L)	0.49*	0.0723	0.0813	0.49*	0.0112	**0.0126**
CRP (mg/L)	0.26*	0.3748	0.3748	−0.01*	0.9722	0.9722
T-AOC (U/mL)	0.72*	0.0038	**0.0114**	0.68*	0.0001	**0.0002**
MDA (nmol/mL)	0.64*	0.0129	**0.0232**	0.75*	<0.0001	**<0.0001**
DHA (ng/mL)	0.54*	0.0449	0.0674	0.71*	0.0001	**0.0002**
EPA (ng/mL)	0.77*	0.0012	**0.0108**	0.76*	<0.0001	**<0.0001**
Uric acid (μmol/L)	0.72*	0.0038	**0.0114**	0.55*	0.0034	**0.0048**
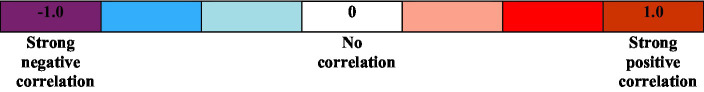						

Ov/Ob, participants with overweight or obesity; Non-Ov/Ob, participants with normal weight; BDNF, brain-derived neurotrophic factor; SFA, saturated fatty acids; MUFA, monounsaturated fatty acids; PUFA, polyunsaturated fatty acids; IL, interleukin; TNF-α, tumour necrosis factor alpha; CRP, C-reactive protein; T-AOC, total antioxidant capacity; MDA, malondialdehyde; DHA, docosahexaenoic acid; EPA, eicosapentaenoic acid. Correlation coefficients are presented as *Spearman’s ρ or **Pearson’s r, depending on data distribution; q = FDR-adjusted *p*-value. Statistically significant associations after FDR correction (*q* < 0.05) are highlighted in bold.

### BDNF and inflammatory, oxidative stress markers, Ω-3 fatty acids, and uric acid

Serum BDNF was positively associated with several inflammatory cytokines across both BMI phenotypes. After FDR correction, IL-1β remained significantly associated with BDNF in both groups, while IL-6 and TNF-*α* remained significant only in normal-weight patients.

Regarding oxidative stress, BDNF showed positive correlations with MDA and total T-AOC in both BMI phenotypes, and these associations remained significant after FDR correction.

Ω-3 fatty acids showed phenotype-dependent associations with BDNF. In normal-weight adolescents, both DHA and EPA remained significantly correlated after FDR correction. In overweight and with obesity adolescents, only EPA remained significantly associated with BDNF, while the association with DHA did not retain significance after correction.

In both BMI phenotypes, BDNF was positively associated with serum uric acid concentrations. Detailed results are presented in Table 2.

### BDNF and physical activity

Serum BDNF levels did not correlate significantly with total physical activity expressed in MET-minutes/week, nor with vigorous, moderate, or walking domains in either BMI group or in the whole cohort. Likewise, comparisons of BDNF concentrations across low, moderate, and high physical activity categories did not reveal significant differences (Table 3). Although a tendency toward higher BDNF levels with increasing physical activity was observed, it did not reach statistical significance (Table 4).

**Table 3:** Correlations between serum BDNF concentrations and physical activity level (MET) in participants with PCOS.

Physical activity	Ov/Ob (*n* = 14)			Non-Ov/Ob (*n* = 26)			All participants (*n* = 40)		
	*r*	*p* value	*q*	*r*	*p* value	*q*	*r*	*p* value	*q*
MET total	0.03	0.9109	0.9642	0.02	0.9168	0.9168	0.05	0.7716	0.8150
MET vigorous	0.11	0.7078	0.9642	−0.12	0.5442	0.7256	−0.04	0.8150	0.8150
MET moderate	−0.01	0.9642	0.9642	0.14	0.5090	0.7256	0.06	0.6995	0.8150
MET walk	0.17	0.5608	0.9642	0.16	0.4375	0.7256	0.17	0.3085	0.8150

Ov/Ob, participants with overweight or obesity; Non-Ov/Ob, participants with normal weight; BDNF, brain-derived neurotrophic factor; MET, metabolic equivalent of task; results expressed as MET·min/week. All correlations were calculated using Spearman’s rank coefficient; q = FDR-adjusted *p*-value.

**Table 4:** Comparison of serum BDNF concentrations across physical activity levels in adolescents with PCOS.

Group	Physical activity level	*n*	BDNF ng/ml, mean ± SD or Median [Q1; Q3]	*p* value
Non-Ov/Ob (*n* = 26)	Low	9	1.17 ± 0.29	0.214*
	Moderate	14	1.15 ± 0.47	
	Vigorous	3	1.62 ± 0.46	
Ov/Ob (*n* = 14)	Low	4	1.58 [1.17; 1.72]	0.744**
	Moderate	5	1.24 [1.09; 1.56]	
	Vigorous	5	2.23 [0.73; 2.85]	
All participants (*n* = 40)	Low	13	1.19 [1.00; 1.54]	0.216**
	Moderate	19	1.17 [0.84; 1.49]	
	Vigorous	8	1.88 [1.00; 2.29]	

Ov/Ob, participants with overweight or obesity; Non-Ov/Ob, participants with normal weight; BDNF, brain-derived neurotrophic factor; MET, metabolic equivalent of task. Physical activity levels were categorized according to total MET values (low, moderate, vigorous). Homogeneity of variances was confirmed for the Non-Ov/Ob group (Levene’s test: *p* = 0.288). *One-way ANOVA, **Kruskal–Wallis test. BDNF, brain-derived neurotrophic factor; MET, metabolic equivalent of task. Physical activity levels were categorized according to total MET values (low, moderate, vigorous). Homogeneity of variances was confirmed for the Non-Ov/Ob group (Levene’s test: *p* = 0.288).

### BDNF in relation to anthropometric indices, age, lipid profile, and glucose-insulin homeostasis

No associations were observed between serum BDNF and age, BMI, or lipid parameters in either subgroup. In normal-weight girls, trends suggested that higher BDNF might be associated with more favourable glucose–insulin homeostasis: BDNF tended to correlate negatively with HOMA-IR and positively with QUICKI, although neither relationship reached statistical significance. These tendencies were absent in patients with overweight and obesity. No significant associations were observed between serum BDNF and age, BMI, lipid parameters, or markers of glucose–insulin homeostasis, including fasting glucose, fasting insulin, homeostasis model assessment of insulin resistance (HOMA-IR), and quantitative insulin sensitivity check index (QUICKI) (Table 5).

**Table 5:** Correlations between serum BDNF concentrations and age, BMI, lipid profile, and insulin sensitivity indices (HOMA-IR, QUICKI) in participants with PCOS.

Parameter	Ov/Ob (*n* = 14)			Non-Ov/Ob (*n* = 26)		
	*r*	*p* value	*q*	*r*	*p* value	*q*
Age (years)	0.23*	0.4318	0.7809	−0.24*	0.2451	0.4902
BMI (kg/m^2^)	0.13*	0.6586	0.7809	0.07**	0.7216	0.8018
Total cholesterol (mg/dL)	−0.14*	0.6261	0.7809	−0.12*	0.5593	0.6991
LDL-C (mg/dL)	−0.14*	0.6369	0.7809	0.05*	0.8048	0.8048
HDL-C (mg/dL)	−0.35*	0.2207	0.7809	−0.19**	0.3669	0.6115
Triglycerides (mg/dL)	0.04*	0.8991	0.8991	−0.13**	0.5365	0.6991
HOMA-IR	0.11*	0.7028	0.7809	−0.35*	0.0835	0.3340
QUICKI	−0.18*	0.5274	0.7809	0.33*	0.1002	0.3340
Fasting glucose (mg/dL)	−0.24*	0.4183	0.7809	−0.29**	0.1534	0.3835
Fasting insulin (μU/ml)	0.15*	0.6048	0.7809	−0.37*	0.0626	0.3340

Ov/Ob, participants with overweight or obesity; Non-Ov/Ob, participants with normal weight; BDNF, brain-derived neurotrophic factor; HDL-C, high-density lipoprotein cholesterol; LDL-C, low-density lipoprotein cholesterol; HOMA-IR, homeostasis model assessment of insulin resistance; QUICKI, quantitative insulin sensitivity check index. Correlation coefficients are presented as *Spearman’s ρ or **Pearson’s r, depending on data distribution; q = FDR-adjusted *p*-value.

### BDNF and sex hormones/cortisol

BDNF was not significantly associated with FSH, LH, estradiol, total and free testosterone, SHBG, DHEA-S, prolactin and cortisol in either phenotype (Table 6).

**Table 6:** Correlations between serum BDNF concentrations and hormone levels in participants with PCOS.

Parameter	Ov/Ob (*n* = 14)			Non-Ov/Ob (*n* = 26)		
	r	*p* value	*q*	*r*	*p* value	*q*
FSH (mIU/mL)	−0.11*	0.7028	0.9228	0.13**	0.5438	0.6798
LH (mIU/mL)	0.31*	0.2882	0.9228	0.05*	0.7945	0.7945
Estradiol (pg/mL)	0.41*	0.1443	0.9228	−0.14*	0.5104	0.6798
Prolactin (ng/mL)	−0.28*	0.3260	0.9228	0.14**	0.5104	0.6798
Total testosterone (nmol/L)	−0.15*	0.6081	0.9228	−0.19**	0.3633	0.6798
Free testosterone (nmol/L)	−0.03*	0.9228	0.9228	−0.17*	0.4047	0.6798
DHEA-S (μg/dL)	−0.05*	0.8755	0.9228	−0.08**	0.7056	0.7840
SHBG (nmol/L)	0.20*	0.4930	0.9228	0.26*	0.2054	0.6798
FAI (T × 100/SHBG)	−0.09*	0.7593	0.9228	−0.18*	0.3877	0.6798
Cortisol (μg/dL)	0.24*	0.4094	0.9228	0.35**	0.0806	0.6798

Ov/Ob, participants with overweight or obesity; Non-Ov/Ob, participants with normal weight; BDNF, brain-derived neurotrophic factor; FSH, follicle-stimulating hormone; LH, luteinizing hormone; DHEA-S, dehydroepiandrosterone sulfate; SHBG, sex hormone-binding globulin; FAI, free androgen index; T, testosterone. Correlation coefficients are presented as *Spearman’s ρ or **Pearson’s r, depending on data distribution; q = FDR-adjusted *p*-value.

## Discussion

The present study demonstrates that circulating BDNF in adolescents with PCOS is associated with inflammatory activity, oxidative–antioxidative balance, and omega-3 fatty acid status across both BMI phenotypes, while dietary associations were observed only in the normal-weight group after FDR correction.

The associations between BDNF and pro-inflammatory cytokines (IL 1β, IL 6, TNF *α*) and oxidative markers (MDA, T-AOC) in both weight groups are consistent with previously described redox-sensitive regulation of BDNF (8, 13, 14). This pattern may reflect a compensatory response to oxidative–inflammatory stress observed in PCOS. The associations with EPA and DHA support lipid-mediated modulation, in line with evidence that omega-3 fatty acids are linked to BDNF regulation and metabolic signaling (15–17).

Uric acid was positively associated with BDNF in both BMI phenotypes. Within the context of the present study, this finding may reflect the clustering of BDNF with redox- and metabolic-related signals rather than an isolated uric acid-specific effect. This interpretation is consistent with the recognized involvement of uric acid in redox and metabolic processes (30, 31). Given the exploratory and cross-sectional design, this association should be interpreted cautiously and warrants verification in larger and longitudinal studies.

A particularly novel finding of this study is the divergence in diet–BDNF associations between weight phenotypes. After FDR correction, significant dietary associations were observed only in normal-weight girls, whereas they were absent in the Ov/Ob group. In the normal-weight group, higher BDNF levels were associated with greater intake of protein, MUFA, and PUFA, nutrients previously linked to metabolic flexibility and neurotrophic signaling (7).

In contrast, in patients with overweight and obesity, no significant dietary associations with BDNF were observed after FDR correction. This may suggest reduced nutrient responsiveness or altered regulation of peripheral BDNF; however, this interpretation should be considered cautiously given the limited sample size and exploratory design. Similar patterns have been suggested in previous studies and have been discussed in the context of chronic inflammation, mitochondrial dysfunction, altered muscle-derived BDNF secretion, or differences in insulin sensitivity (32–34).

These phenotype-dependent differences are consistent with the metabolic heterogeneity of PCOS and may have implications for individualized nutritional approaches. Associations between circulating BDNF and cardiometabolic parameters, including lipid metabolism, have also been reported in adolescent populations (35).

The absence of significant associations between BDNF and physical activity contrasts with findings from structured exercise interventions (22, 23, 36). This discrepancy may reflect the limitations of self-reported IPAQ-SF measures, as well as the relatively narrow variability of habitual physical activity within this adolescent cohort, which may have reduced statistical power to detect associations. Similarly, the lack of associations with sex hormones is consistent with reports that adolescent neuroendocrine systems remain in transition, and that inflammatory and metabolic factors may exert stronger influences on circulating BDNF during this developmental period (37, 38). Hormonal regulation of BDNF has nevertheless been demonstrated in experimental models, particularly in relation to androgen signaling and neuroplasticity (39, 40).

The positive trend between BDNF and cortisol aligns with hypothalamic–pituitary–adrenal (HPA) axis responses described in stress physiology (41–45). In this context, the observed tendency may indicate that neurotrophic regulation in adolescent PCOS is also influenced by stress-related neuroendocrine activity; however, this relationship did not remain significant after multiple testing correction and should be interpreted with caution.

Overall, the observed association patterns suggest that circulating BDNF may be linked to nutritional, inflammatory, and oxidative domains in adolescent PCOS, with limited evidence for endocrine associations. These findings should be interpreted as exploratory.

## Conclusion

In adolescent girls with PCOS, circulating BDNF was associated with dietary quality, inflammatory activity, oxidative–antioxidative balance, omega-3 fatty acid status, and selected metabolic parameters, with limited evidence for endocrine associations, whereas no association with adiposity per se was observed. The phenotype-dependent dietary associations suggest that the relationship between BDNF and nutritional factors may differ across metabolic contexts. Given the cross-sectional design and exploratory nature of the analyses, these findings should be considered hypothesis-generating.

Future longitudinal and interventional studies are needed to determine whether BDNF may have clinical relevance in adolescent PCOS, including its potential role as a marker reflecting metabolic and inflammatory profiles and response to nutritional and anti-inflammatory interventions.

### Study limitations

This study has several limitations that should be considered when interpreting the findings. The relatively small sample size (*n* = 40) and recruitment from a single clinical centre may limit the generalizability of the results. The lack of a healthy control group precludes determining whether the observed BDNF associations are specific to PCOS or reflect broader metabolic patterns in adolescent females.

The cross-sectional design precludes causal inferences regarding the relationships between BDNF, dietary patterns, inflammatory activity, oxidative–antioxidative balance, metabolic parameters, and cortisol. Dietary intake was assessed using 3-day food records, which provide short-term estimates of intake but may not fully reflect habitual dietary patterns due to day-to-day variability Physical activity was assessed using self-reported questionnaires, which are susceptible to recall bias and may not fully capture habitual behaviors or variability over time. Moreover, physical activity was evaluated under free-living conditions rather than under controlled exercise settings, which may have contributed to the lack of observed associations with BDNF.

Although key hormonal, metabolic, inflammatory, and oxidative markers were included, several relevant factors known to influence neurotrophic regulation—such as psychosocial stress, sleep quality, and gut microbiota-related pathways—were not assessed in the present study. These factors may interact with inflammatory and metabolic processes and could contribute to variability in circulating BDNF and related immunometabolic parameters (46–49).

Furthermore, BDNF was measured only in serum, without assessment of tissue-specific expression or differentiation between mature BDNF and its precursor proBDNF, which may have distinct physiological roles. The ELISA used quantifies total BDNF and does not distinguish BDNF isoforms, and serum concentrations may also be influenced by platelet content and activation, which were not evaluated. Although blood sampling was standardized to morning hours after overnight fasting, diurnal variation in circulating BDNF and cortisol cannot be fully excluded. Although body composition variables (FM, FFM, and muscle mass) were assessed, we did not directly evaluate muscle activity-dependent BDNF secretion or distinguish tissue-specific sources of circulating BDNF. Oxidative stress assessment was limited to MDA and T-AOC, whereas more specific biomarkers, such as F2-isoprostanes, were not included. Finally, although a non-significant tendency between BDNF and cortisol was observed, detailed assessment of HPA axis activity—such as ACTH levels or dynamic cortisol profiling—was not performed, restricting the interpretation of stress-related neuroendocrine influences on neurotrophic regulation.

## Data Availability

The raw data supporting the conclusions of this article will be made available by the authors, without undue reservation.

## Footnotes

1
www.myassays.com
